# Supplementary material for: SARS-CoV-2 Antibody Response against Mild-to-Moderate Breakthrough COVID-19 in Home Isolation Setting in Thailand
Source: Vaccines (Basel). 2022 Jul 15;10(7):1131. doi: 10.3390/vaccines10071131 (PMC9318962; doi:10.3390/vaccines10071131)
Supplement: Supplementary file 1 [file vaccines-10-01131-s001.zip › vaccines-1795631-supplementary.pdf]

Supplementary Appendix

Supplement to: SARS-CoV-2 antibody response against mild-to-moderate breakthrough COVID-19 in home isolation setting in Thailand

This appendix has been provided by the authors to give readers additional information about the work.

Contents: **Page**

Investigators and support team members	3
Figure S1: Trend of SARS-CoV-2 variants circulating in Thailand, deaths and vaccination status during analysis period	5
Methods S1: Study design, eligibility, and inclusion and exclusion criteria	6
Table S1: General comorbidity questionnaire	10
Table S2: Characteristics of participants at enrollment (Jul-Oct 2021 (Delta)) by age	13
Table S3: Characteristics of participants at enrollment (Jan-Mar 2022 (Omicron)) by age	16
Table S4: Independent factors of NCT of SARS-CoV-2 RNA by the multivariate Cox's proportional hazard model by age >18 years (Jul-Oct 2021 (Delta))	19
Figure S2: Kaplan-Meier estimate of time to symptom alleviation as reported by COVID-19-adapted FLU-PRO questionnaire (Jul-Oct 2021 (Delta))	20
Table S5: Characteristics of the first positive symptoms between the Delta and Omicron pandemics for people aged >18 years	21
Table S6: Independent associations with symptoms reporting between the Delta and Omicron pandemics for people aged >18 years	22
Table S7: Characteristics of the participants and geometric mean humoral immunogenicity assay responses according to anti-SARS-CoV-2 nucleocapsid protein and vaccine group	23
Table S8: Anti-RBD IgG and sVNT geometric mean concentration (GMC) among unvaccinated, ChAdOx1, and CoronaVac	26
Table S9: SARS-CoV-2 sVNT geometric mean concentration (GMC) among unvaccinated, ChAdOx1, and CoronaVac by Wuhan and Delta variants	27
Table S10: Anti-RBD IgG and sVNT geometric mean concentration (GMC) among unvaccinated, ChAdOx1, and CoronaVac by age	28
Figure S3: Plasma anti-immunoglobulin G (IgG) and antibody (sVNT) levels against SARS-Cov-2	29

Investigators and Support Team Members

SI-Home Study Team

This project authors gratefully express our enormous gratitude to all members of SPHERE staff members and research assistants as well as all staff of Siriraj Hospital who work tirelessly to take care of individuals with COVID-19. This study and work done would not have been finished without their dedications and efforts. We also would like to acknowledge Associate Professor. Visit Vamvanij, the director of Siriraj Hospital for his guidance for initiation of this project.

SI-Home study is a multidisciplinary working group of Siriraj Hospital to together implement appropriate public health interventions in response to COVID-19. These actions include active and passive surveillance, active case finding, contact tracing, preparation of resource, laboratory capacity to diagnose SARS-CoV-2 infection, quarantine facilities and services, etc. The emphasis is on efficient response with appropriate target populations.

Siriraj Executive Committee

Prasit Wattanapa[1], Visit Vamvanij[2], Chamaiporn Charoenkraikamol[3], Daranee Piputtanakulchai[3]

[1]Dean, Faculty of Medicine Siriraj Hospital, Mahidol University; [2]Director, Faculty of Medicine Siriraj Hospital, Mahidol University; [3]Department of Nursing, Faculty of Medicine Siriraj Hospital, Mahidol University

Medical Monitoring Team

Pochamana Phisalprapa[1], Naruemit Sayabovorn[1], Weerachai Srivanichakorn[1], Chaiwat Washirasaksiri[1], Chonticha Auesomwang[1], Tullaya Sitasuwan[1], Thanet Chaisathaphol[1], Rungsima Tinmanee[1], Methee Chayakulkeeree[2], Pakpoom Phoompoung[2], Gornmigar, Sanpawitayakul[3], Rungsima Wanitphakdeedecha[4], Saipin Muangman[5], Korapat Mayurasakorn[6]

[1]Division of Ambulatory Medicine, Department of Medicine, Faculty of Medicine Siriraj Hospital, Mahidol University; [2]Division of Infectious Diseases and Tropical Medicine, Department of Medicine, Faculty of Medicine Siriraj Hospital, Mahidol University; [3]Department of Pediatrics, Faculty of Medicine Siriraj Hospital, Mahidol University; [4]Department of Dermatology, Faculty of Medicine Siriraj Hospital, Mahidol University; [5]Department of Anesthesiology, Faculty of Medicine Siriraj Hospital, Mahidol University; [6]Siriraj Population Health and Nutrition Research Group (SPHERE), Department of Research Group and Research Network, Siriraj Medical Research Center, Mahidol University

Si-Home Management Team

Sureeporn Pumeiam[1] (coordinator), Bonggochpass Pinsawas[1] (online coordinator), Pichanun Mongkolsucharitkul[1] (online coordinator), Apinya Surawit[1] (data analyzer), Tanyaporn Pongkunakorn[1] (online coordinator), Sophida Suta[1] (online coordinator), Thamonwan Manosan[1] (online coordinator), Suphawan Ophakas[1] (online coordinator), Nattvara Nirdnoy[1] (online coordinator), Korapat Mayurasakorn[1] (online supervisor), Suree Leemongkol(continuing care manager)[2], Usanee Fongsri[2], Pranom Sahunphun[2], Panita Janthapadchothe[3]

[1]Siriraj Population Health and Nutrition Research Group (SPHERE), Department of Research Group and Research Network, Siriraj Medical Research Center, Mahidol University; [2]Department of Nursing, Faculty of Medicine Siriraj Hospital, Mahidol University; [3]Pharmacy Department, Faculty of Medicine Siriraj Hospital, Mahidol University

SI-Home Operational Team

Penrapee Phoosongtham[1] (site manager), Wanwalee Kochasawas[1] (nurse case manager), Phakamas Jitpun[1] (charge nurse)

[1]Department of Nursing, Faculty of Medicine Siriraj Hospital, Mahidol University

Trend of SARS-CoV-2 variants circulating in Thailand

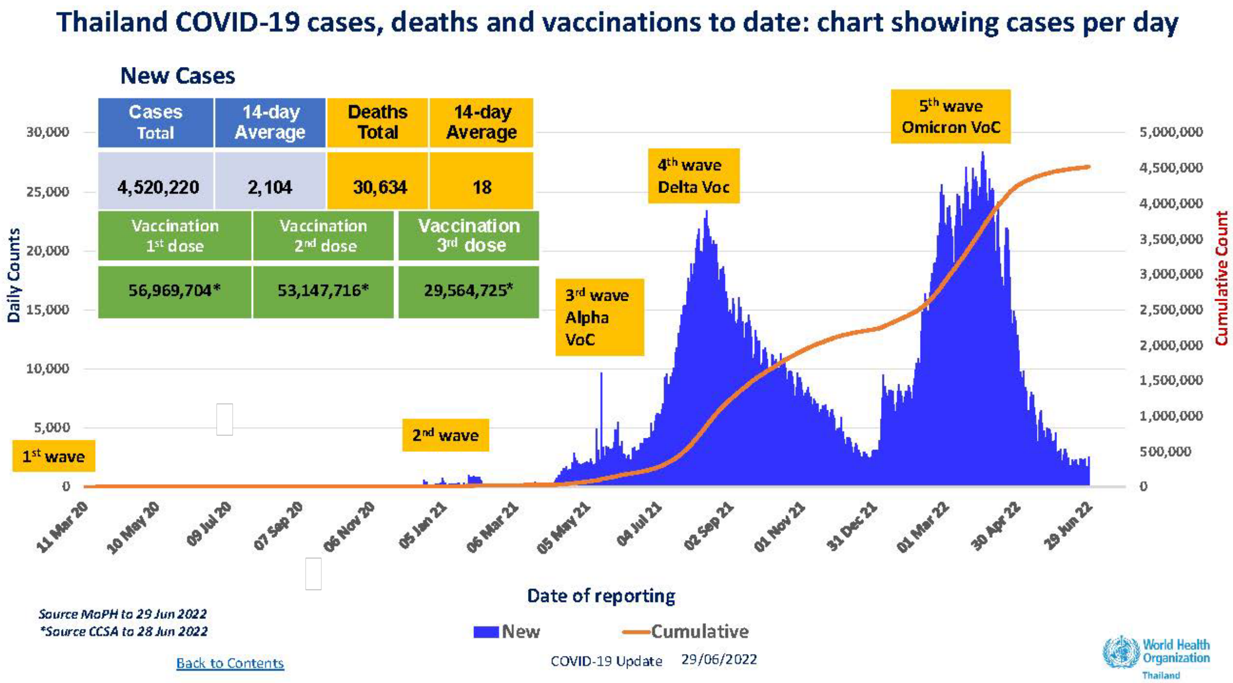

Figure S1. Trend of SARS-CoV-2 variants circulating in Thailand, deaths and vaccination status during analysis period [1]

The average number of new cases and death per million population are decreasing across provinces, with lower average rates being reported far more widely across Thailand compared to the previous two weeks. Most cases continue to be monitored in hospitals and satellite facilities (hospitels, community isolation and outpatient home isolation). The average number of COVID-19 cases occupying hospital beds per day over the past 14 days decreases by 13.4%. Thai health authorities are closely monitoring the potential new surge of Omicron subvariants BA.4/BA.5 that are becoming dominant COVID-19 strains globally. These two subvariants were reported to make up around 40% of new COVID-19 cases in Thailand. Vaccination rates in Thailand continue to significantly reduce levels of severe illness and deaths caused by circulating Omicron variants. High vaccination rates also help ameliorate the transmission of COVID-19.

Methods S1. Study design, eligibility, and inclusion and exclusion criteria

Study design

This was a multidisciplinary, retrospective cohort study involving COVID-19 patients admitted at HI system and monitored by remote medical consultation in Bangkok, Thailand. A total of 11,897 patients with mild to moderate COVID-19 were isolated at home in Siriraj HI system during July 8, 2021 to May 11, 2022. The data collection reported here was performed between July 8, 2021 to March 15, 2022. The study population included 2704 and 2477 patients during the Delta (before November 2021) and Omicron (after January 12, 2022) pandemic who had been positive with SARS-CoV-2 as determined by a reverse transcriptase polymerase chain reaction (RT-PCR) testing. Someone who met inclusion criteria would be considered to have mild symptoms, or perhaps be asymptomatic, and would be referred to Siriraj-Home system (SI-Home) where medicine will be delivered by health personnel within 24 hours rather than being relegated to a field hospital or other potentially unpleasant arrangement. A subset of 495 patients during Delta pandemic (July-October 2021) were followed for serology testing between 21-120 days post COVID-19 onset. This study was approved by the Institutional Review Board of the Faculty of Medicine Siriraj Hospital, Mahidol University (SIRB protocol 463/2564; COA: Si 503/2021 and SIRB protocol 769/2564; COA: Si 833/2021).

Inclusion criteria for those considered as COVID-19 infection with mild-to-moderate conditions suitable for SI-Home

1. Age ≥12 years with any sexs
2. Well-controlled chronic underlying diseases (e.g. dyslipidemia, diabetes mellitus, hypertension)
3. Body temperature: below 39 degrees Celsius during PCR testing day
4. Able to use online communication platforms
5. Oxygen saturation (more than a 95% oxygen saturation value)
6. No evidence of pneumonia on an admission date
7. All patients in HI system were subject to a maximum of 10-14 days home quarantine according to a governmental guideline [2]

Criteria for referral

1. Anyone with a fever that stays above 39 degrees Celsius for more than 24 hours
2. Anyone with less than a 94% oxygen saturation value
3. Any adult who is inhaling faster than 25 breaths per minute

4. Children who have difficulty breathing or experience rapid breathing, loss of appetite, drowsiness, as well as a fever over 39 degrees Celsius and an oxygen saturation value under 94%

Testing and monitoring processes

All patients in this study were admitted in HI system. During isolation in the HI, patients had their temperatures and respiratory symptoms checked ≥2 times each day, either by telemedicine with nurses or by using self-monitoring equipment and reported via an electronic diary. Online (24 hours/7 days) communication with medical staff was a standard management to monitor progression of the COVID-19 for medical management. Customized sets of medication would be provided for each patient for symptomatic treatment, such as antipyretics antitussives and cetirizine, which were prescribed by a medical staff via an online platform. Online medical staff included infectious disease physicians, infectious pediatricians, ambulatory physicians, psychiatrists, family physicians, obstetricians, registered nurses, volunteers, adverse drug reaction monitoring pharmacists, dietitians and the referral center staff. Breathing exercises with prone position was a recommended technique for patients with dyspnea and reduced oxygen saturation. Sit-to-stand tests for exercise-induced desaturation was one of the techniques to verify a so-called "happy hypoxia" [3]. Patients who developed symptoms such as dyspnea, chest pain, or chest discomforts, which did not subside after recommended treatment were transferred back to the hospital.

The days of post symptom onset (PSO) was determined based on the difference between the date of the RT-PCR testing and the date of first reported symptoms coherent with COVID-19. For asymptomatic individuals, the day from the first positive SARS-CoV-2 PCR testing was used. The durations of illness onset to first hospital admission, to first Favipiravir treatment and to discharge up to 14 days were measured. Favipiravir was recommended anti-viral therapy especially in patients with high risk for COVID-19 pneumonia within 48 hours and was given for 5 days, but duration can be extended to as long as 14 days based on patient's clinical severity. The dose was 1800 mg twice daily on the first day followed by 800 mg twice daily [4].

A subpopulation follow-up study

A subset of 495 participants during the Delta pandemic provided general information, local and systemic symptoms, and a vaccination history. Vaccine history of participants, including type, number of doses, and date(s) of vaccination, as described in the COVID-19 Application Privacy Notice ("Morprom"), if available. Additional blood samples were collected via phlebotomy in ethylenediaminetetraacetic acid (EDTA) tubes and processed for plasma between 21-120 days post COVID-19 onset. All sera were obtained

and stored at -80 °C within Siriraj Biobank, Department of Research, Siriraj Medical Research Center, Mahidol University. All data, including demographic, clinical, laboratory, and therapy details were collected from a database of HI system after July 2021. Participants who were vaccinated after being discharged from HI were excluded. Additional plasma samples were collected.

At the study visit, all patients were classified into one of different exposure groups based on vaccination status prior to COVID-19 infection, study antibody and PCR test. Groups were included (1) patients who had not had any vaccination, (2) patients who had a first vaccination <14 days before infection, (3) patients who had a first vaccination ≥14 days before infection, (4) patients who had a second vaccination <14 days before infection, (5) patients who had a second vaccination ≥14 days before infection, and on rare occasion had (6) patients who had a third vaccination <14 days before infection, and (7) patients who had a third vaccination ≥14 days before infection.

SARS-CoV-2 PCR testing

Our COVID-19 diagnostic assay was a probe-based qualitative RT-PCR probe. Allplex™ 2019-nCoV Assay (Seegene, Seoul, South Korea) was used for SARS-CoV2 detection. The targeted COVID-19 genes detected here included nucleocapsid (N), envelope (E) of Sarbecovirus and RNA-dependent RNA polymerase (RdRp) of COVID-19 according to the manufacturer's instructions and described previously [5].

Outcomes and statistical analysis

The characteristics of the patients at baseline were reported as counts and percentages or, for continuous variables, as medians with interquartile ranges. For the descriptive analysis, categorical variables were presented as absolute numbers and their relative frequencies. Continuous variables were summarized as mean and standard deviation if normally distributed or as the median and interquartile range (IQR) if not normally distributed. Comparisons of Ct values by vaccine exposure groups used quantile (median) regression adjusted for age and sex (Figure 2) in full text of this article. Patients who responded to the questionnaire were included in the analysis, and results are presented as percentages with means or medians and 95% confidence interval (CI). In univariable analysis, categorical variables were compared using the chi-square test and binomial logistic regression and presented with ORs, 95% CI and *p*-values. Multivariable analysis was performed by binary logistic regression for dichotomous outcome variables (Tables S5-6). Cox regression analysis was used to analyze the factors of the negative conversion time (NCT) of SARS-CoV-2 RNA (NCT is closely related to clinical manifestation and disease progression

in COVID-19 patients. First, univariate analysis was performed, and the indicators with statistical significance were analyzed for Kaplan-Meier survival analysis. A Cox proportional hazard model was used for multivariate analysis (Table S4 and Figure S2). The differences in continuous and categorical data were tested using the Mann-Witney U test or t-test, Kruskal-Wallis H test or Analysis of variance (ANOVA) and chi-square test, respectively. The anti-SARS-CoV-2 RBD IgG concentration and neutralization antibodies were reported as geometric mean titers (GMT) with 95% CI. Unpaired t test, and ANOVA were used to compare GMT within group, between groups, and across groups using GraphPad Prism 9 version 9.2.0 (GraphPad Software, CA, USA), respectively (Tables S7-10 and Figure S4). All analyses were conducted in STATA version 17 (Stata Corp, LP, College Station, TX, USA), and graphs were produced in GraphPad Prism 9 (GraphPad Software, CA, USA).

Table S1. General comorbidity questionnaire

Questions	Answer 1	Answer 2	Answer 3
**General information**			
1. Age		________ years	
2. Sex	Male	Female	
3. Pregnancy status	Yes (___ weeks)	No	
4 Career		__________	
5. Do you have any comorbidities? (chronic upper respiratory tract disease, cardiovascular disease, chronic kidney disease, cerebrovascular disease, obesity, cancer, uncontrolled diabetes)	Yes	No	
6. Did you experience COVID-19 infection in the previous 3 months?	Yes	No	Not sure
7. Did you have a history of COVID-19 reinfection?	Yes	No	Not sure
**Evaluation of previous health status**			
8. Did you experience significant weight loss?	Yes	No	Not sure
9. Did you experience anorexia and nausea?	Yes	No	Not sure
10. Did you experience erectile dysfunction?	Yes	No	Not sure
11. Did you experience menstrual changes?	Yes	No	Not sure
12. Did you experience being unable to control movement?	Yes	No	Not sure
13. Did you experience feeling of a numb body or face?	Yes	No	Not sure
14. Did you experience stability issues?	Yes	No	Not sure
15. Did you experience dysphagia problems?	Yes	No	Not sure
16. Did you experience vision problems?	Yes	No	Not sure
17. Did you experience communication problems?	Yes	No	Not sure
18. Did you experience sleep problems?	Yes	No	Not sure
19. Did you experience urinary tract problems?	Yes	No	Not sure
**Evaluation of adverse signs and symptoms in the past 7 days**			
20. Did you experience fever?	Yes	No	Not sure
21. Did you experience headache?	Yes	No	Not sure
22. Did you experience cough?	Yes	No	Not sure

Table S1. General comorbidity questionnaire

23.	Did you experience sore throat?	Yes	No	Not sure
24.	Did you experience running nose?	Yes	No	Not sure
25.	Did you experience shortness of breath or difficulty breathing?	Yes	No	Not sure
26.	Did you experience chest pain?	Yes	No	Not sure
27.	Did you experience myalgia or weakness?	Yes	No	Not sure
28.	Did you experience joint pain?	Yes	No	Not sure
29.	Did you experience fatigue?	Yes	No	Not sure
30.	Did you experience leg/arm numbness?	Yes	No	Not sure
31.	Did you experience confusion or stupor?	Yes	No	Not sure
32.	Did you experience any seizure?	Yes	No	Not sure
33.	Did you experience abdominal pain or diarrhea?	Yes	No	Not sure
34.	Did you experience nausea, diarrhea or vomiting?	Yes	No	Not sure
35.	Did you experience conjunctivitis or blurred vision?	Yes	No	Not sure
36.	Did you experience rash?	Yes	No	Not sure
**COVID-19 vaccination histories**				
37.	Did you get any COVID-19 vaccines before?	Yes	No	Not sure
38.	How many shots of COVID-19 vaccine?		___________ shot(s)	
39.	Types of COVID-19 vaccination (Sinovac, AstraZeneca, Sinopharm, Pfizer, Moderna, Johnson& Johnson, Novavax, Sputnik V)		____________	
40.	Did you experience an allergic reaction? If so, please explain.	Yes	No	Not sure
41.	Did you experience other symptoms after receiving vaccines? If so, please explain.	Yes	No	Not sure
**Symptoms during COVID-19 care in HI system**				
1.	Did you experience fever?	Yes	No	Not sure
2.	Did you experience cough?	Yes	No	Not sure
3.	Did you experience sore throat?	Yes	No	Not sure
4.	Did you experience Rhinorrhea?	Yes	No	Not sure
5.	Did you experience Productive sputum?	Yes	No	Not sure

Table S1. General comorbidity questionnaire

6.	Did you experience Loss of taste?	Yes	No	Not sure
7.	Did you experience Loss of smell?	Yes	No	Not sure
8.	Did you experience Dyspnea?	Yes	No	Not sure
9.	Did you experience Myalgia?	Yes	No	Not sure
10.	Did you experience Diarrhea?	Yes	No	Not sure
11.	Did you experience Nausea/vomiting?	Yes	No	Not sure
12.	Did you experience Others?	Yes	No	Not sure

**Questions of chalder fatigue scale***	**less than usual (0)**	**no more than usual (1)**	**more than usual (2)**	**much more than usual (3)**
1. Do you have problems with tiredness?	☐	☐	☐	☐
2. Do you feel sleepy or drowsy?	☐	☐	☐	☐
3. Do you lack energy?	☐	☐	☐	☐
4. Do you have less strength in your muscles?	☐	☐	☐	☐
5. Do you feel weak?	☐	☐	☐	☐
6. Do you have difficulties concentrating?	☐	☐	☐	☐
7. Do you need to rest more?	☐	☐	☐	☐
8. Do you have problems starting things?	☐	☐	☐	☐
9. How is your memory?	☐	☐	☐	☐

*This scale can be scored "bimodally" with columns representing 0 & 1 and a range from 0 to 9 with a total of 4 or more qualifying for "caseness". Alternatively, it can be stored in "Likert" style 0, 1, 2 & 3 with a range from 0 to 27.

Table S2. Characteristics of participants at enrollment (Jul-Oct 2021 (Delta)) by age

Characteristics	Jul-Oct 2021 (Delta)													
	**All patients**		**0-11 years**		**12-18 years**		**19-30 years**		**31-45 years**		**46-60 years**		**>60 years**	
	**n**	**%**	**n**	**%**	**n**	**%**	**n**	**%**	**n**	**%**	**n**	**%**	**n**	**%**
	**n=2704**		**n=448**		**n=227**		**n=560**		**n=677**		**n=513**		**n=279**	
Age, years, mean (SD)	33.8	(19.6)	5.2	(3.4)	15.2	(2.1)	24.7	(3.3)	38.3	(4.4)	52.8	(4.4)	67.0	(5.6)
Sex														
male	1253	46.3	221	49.3	115	50.7	266	47.5	318	47.0	225	43.9	108	38.7
female	1451	53.7	227	50.7	112	49.3	294	52.5	359	53.0	288	56.1	171	61.3
Body weight, kg, median (IQR)	57.5	(22.0)	23.2	(13.6)	56.4	(18.6)	65.9	(17.3)	67.1	(14.8)	65.9	(13.6)	62.6	(11.7)
Presence of comorbidities														
Diabetes mellitus	162	6.0	0	0.0	0	0.0	3	0.5	19	2.8	67	13.1	73	26.2
Hypertension	313	11.6	0	0.0	0	0.0	9	1.6	48	7.1	118	23.0	138	49.5
Dyslipidemia	123	4.6	0	0.0	0	0.0	1	0.2	18	2.7	46	9.0	58	20.8
Obesity	23	0.9	2	0.5	4	1.8	4	0.7	8	1.2	3	0.6	2	0.7
Malignancy	21	0.8	0	0.0	0	0.0	3	0.5	4	0.6	6	1.2	8	2.9
Neurologic disease	7	0.3	0	0.0	0	0.0	2	0.4	1	0.2	4	0.8	0	0.0
Heart disease	33	1.2	2	0.5	1	0.4	1	0.2	5	0.7	13	2.5	11	3.9
Lung disease	51	1.9	1	0.2	0	0.0	2	0.4	9	1.3	19	3.7	20	7.2
Kidney disease	14	0.5	0	0.0	0	0.0	2	0.4	3	0.4	3	0.6	6	2.2
Others	336	12.4	43	9.6	22	9.7	57	10.2	90	13.3	84	16.4	40	14.3
Dead after referral	5	0.2	0	0.0	0	0.0	0	0.0	0	0.0	3	0.6	2	0.7
Presenting symptoms														
Asymptomatic infection	390	14.4	99	22.1	32	14.1	42	7.5	87	12.9	79	15.4	51	18.3
Fever/history of fever	1250	46.2	262	58.5	96	42.3	261	46.6	305	45.1	208	40.6	118	42.3
BT(°C), median (IQR)	36.6	(0.7)	36.3	(1.0)	36.3	(0.6)	36.7	(0.5)	36.7	(0.5)	36.7	(0.5)	36.7	(0.8)
<37.5	2474	95.2	387	93.3	203	96.2	514	95.0	632	96.1	476	94.8	262	96.0
37.5-38.0	120	4.6	26	6.3	8	3.8	27	5.0	26	4.0	26	5.2	7	2.6

Table S2. Characteristics of participants at enrollment (Jul-Oct 2021 (Delta)) by age

Characteristics	Jul-Oct 2021 (Delta)													
	All patients		0-11 years		12-18 years		19-30 years		31-45 years		46-60 years		>60 years	
	n	%	n	%	n	%	n	%	n	%	n	%	n	%
	n=2704		n=448		n=227		n=560		n=677		n=513		n=279	
>38.0	6	0.2	2	0.5	0	0.0	0	0.0	0	0.0	0	0.0	4	1.5
Cough	1642	60.7	212	47.3	128	56.4	354	63.2	438	64.7	332	64.7	178	63.8
Sore throat	1038	38.4	63	14.1	82	36.1	286	51.1	303	44.8	208	40.6	96	34.4
Rhinorrhea	419	15.5	56	12.5	42	18.5	109	19.5	120	17.7	55	10.7	37	13.3
Productive sputum	537	19.9	40	8.9	45	19.8	140	25.0	170	25.1	88	17.2	54	19.4
Loss of taste	312	11.5	7	1.6	35	15.4	110	19.6	99	14.6	46	9.0	15	5.4
Loss of smell	821	30.4	21	4.7	88	38.8	266	47.5	263	38.9	145	28.3	38	13.6
Dyspnea	305	11.3	6	1.3	18	7.9	76	13.6	88	13.0	73	14.2	44	15.8
Myalgia (muscle aches)	282	10.4	5	1.1	22	9.7	70	12.5	108	16.0	47	9.2	30	10.8
Diarrhea	126	4.7	11	2.5	16	7.1	40	7.1	39	5.8	11	2.1	9	3.2
Nausea/vomiting	59	2.2	4	0.9	3	1.3	12	2.1	24	3.6	8	1.6	8	2.9
Others	1549	57.3	218	48.7	126	55.5	351	62.7	405	59.8	285	55.6	164	58.8
Clinical features at the time of admission														
Time from symptom onset to PCR diagnosis, days, median (IQR)	1.9	(1.6)	2.2	(2.4)	1.9	(1.6)	2.1	(1.5)	1.9	(1.4)	1.6	(1.1)	1.8	(1.6)
Time from symptom onset to admission, days, median (IQR)	5.1	(2.4)	4.8	(2.6)	5.4	(2.6)	5.1	(2.2)	5.1	(2.3)	5.0	(2.3)	5.0	(2.3)
Cycle Threshold, median (IQR)														
Nucleocapsid (N)	21.0	(7.8)	20.1	(7.9)	21.7	(8.9)	21.8	(7.4)	21.1	(8.9)	21.0	(7.3)	20.0	(7.2)
<20	1118	43.2	219	49.7	81	36.0	204	38.1	267	42.3	213	43.4	134	50.4
20-30	1218	47.0	174	39.5	117	52.0	284	53.0	301	47.6	230	46.8	112	42.1
>30	255	9.8	48	10.9	27	12.0	48	9.0	64	10.1	48	9.8	20	7.5
Envelope (E)	17.5	(8.4)	16.9	(8.4)	18.4	(9.2)	18.3	(7.9)	17.7	(9.6)	17.5	(7.7)	16.3	(7.9)

Table S2. Characteristics of participants at enrollment (Jul-Oct 2021 (Delta)) by age

**Characteristics**	**Jul-Oct 2021 (Delta)**													
	**All patients**		**0-11 years**		**12-18 years**		**19-30 years**		**31-45 years**		**46-60 years**		**>60 years**	
	**n**	**%**	**n**	**%**	**n**	**%**	**n**	**%**	**n**	**%**	**n**	**%**	**n**	**%**
	**n=2704**		**n=448**		**n=227**		**n=560**		**n=677**		**n=513**		**n=279**	
<20	1713	64.7	292	66.7	135	60.5	344	62.4	415	62.2	341	67.5	186	70.2
20-30	838	31.6	134	30.6	81	36.3	187	33.9	225	33.7	140	27.7	71	26.8
>30	98	3.7	12	2.7	7	3.1	20	3.6	27	4.1	24	4.8	8	3.0
RNA-dependent RNA polymerase (RdRp)	22.3	(8.2)	21.8	(7.9)	23.4	(9.2)	23.2	(7.5)	22.3	(9.1)	22.1	(7.3)	21.2	(7.6)
Immunosuppression, median (IQR)	1060.6	(1070.1)	-	-	762	(730.5)	1794.9	(1667.8)	805.5	(745.7)	1606.9	(1481.9)	1224.3	(1192.6)
Neutralizing antibody titers, median (IQR)	96.8	(50.9)	-	-	82.2	(74.7)	85.2	(83.4)	91.8	(61.7)	94.3	(36.1)	90.9	(30.0)

Physical symptoms information and laboratory analysis results of COVID-19 patients in the Delta wave by age range. Data are presented as mean (SD), median (IQR), and range at $p < 0.05$ indicates statistical significance. SD, standard deviation; IQR, interquartile range; BT, body temperature; °C, degree Celsius, SARS-CoV-2 spike protein antibody titers, $\log_{10}$ transformed. Neutralizing antibody titers, $\log_{10}$ transformed.

Table S3. Characteristics of participants at enrollment (Jan-Mar 2022 (Omicron)) by age

Characteristics	Jan-Mar 2022 (Omicron)													
	All patients		0-11 years		12-18 years		19-30 years		31-45 years		46-60 years		>60 years	
	n	%	n	%	n	%	n	%	n	%	n	%	n	%
	n=2447		n=516		n=234		n=544		n=518		n=360		n=242	
Age, years, mean (SD)	31.3	(20.3)	5.6	(3.5)	14.9	(1.8)	24.8	(3.3)	37.8	(4.4)	52.4	(4.4)	69.8	(7.4)
Sex														
male	1031	41.6	269	52.1	117	50.0	203	37.3	202	34.8	137	38.1	103	42.6
female	1446	58.4	247	47.9	117	50.0	341	62.7	379	65.2	223	61.9	139	57.4
Body weight, kg, median (IQR)	55.8	(22.4)	24.7	(16.1)	59.0	(18.4)	62.4	(16.0)	66.7	(16.1)	65.9	(12.8)	61.1	(13.6)
Presence of comorbidities														
Diabetes mellitus	150	6.1	0	0.0	1	0.4	2	0.4	25	4.8	49	13.6	73	30.2
Hypertension	513	20.7	32	6.2	25	10.7	63	11.6	108	20.9	140	38.9	145	59.9
Dyslipidemia	108	4.4	0	0.0	0	0.0	0	0.0	19	3.7	35	9.7	54	22.3
Obesity	41	1.7	5	1.0	6	2.6	5	0.9	13	2.5	11	3.1	1	0.4
Malignancy	25	1.0	0	0.0	0	0.0	0	0.0	0	0.0	10	2.8	15	6.2
Neurologic disease	271	10.9	12	2.3	20	8.6	95	17.5	94	18.2	37	10.3	13	5.4
Heart disease	38	1.5	3	0.6	0	0.0	1	0.2	8	1.5	10	2.8	16	6.6
Lung disease	na		na		na		na		na		na		na	
Kidney disease	10	0.4	1	0.2	0	0.0	0	0.0	1	0.2	6	1.7	2	0.8
Others	753	30.4	75	14.5	44	18.8	90	16.5	169	32.6	191	53.1	184	76.0
Dead after referral	2	0.1	0	0.0	0	0.0	0	0.0	0	0.0	0	0.0	2	0.8
Presenting symptoms														
Asymptomatic infection	969	39.1	266	51.6	99	42.3	167	30.7	183	35.3	142	39.4	112	46.3
Fever/history of fever	267	10.8	156	30.2	35	15.0	31	5.7	19	3.7	12	3.3	14	5.8
BT (°C), median (IQR)	36.8	(0.5)	36.9	(1.3)	36.8	(0.8)	36.8	(0.4)	36.7	(0.4)	36.7	(0.3)	36.8	(0.4)

Table S3. Characteristics of participants at enrollment (Jan-Mar 2022 (Omicron)) by age

Characteristics	Jan-Mar 2022 (Omicron)													
	**All patients**		**0-11 years**		**12-18 years**		**19-30 years**		**31-45 years**		**46-60 years**		**>60 years**	
	**n**	**%**	**n**	**%**	**n**	**%**	**n**	**%**	**n**	**%**	**n**	**%**	**n**	**%**
	**n=2447**		**n=516**		**n=234**		**n=544**		**n=518**		**n=360**		**n=242**	
<37.5	2107	88.2	317	64.8	172	71.1	509	88.2	557	90.9	342	87.5	210	80.5
37.5-38.0	244	10.2	135	27.6	33	13.6	31	5.4	19	3.1	12	3.1	14	5.4
>38.0	23	1.0	21	4.3	2	0.8	0	0.0	0	0.0	0	0.0	0	0.0
Cough	1181	47.7	163	31.6	107	45.7	319	58.6	322	62.2	168	46.7	102	42.2
Sore throat	1181	47.7	163	31.6	107	45.7	319	58.6	322	62.2	168	46.7	102	42.2
Rhinorrhea	626	25.3	85	16.5	56	23.9	165	30.3	181	34.9	89	24.7	50	20.7
Productive sputum	na		na		na		na		na		na		na	
Loss of taste	43	1.7	0	0.0	2	0.9	17	3.1	19	3.7	5	1.4	0	0.0
Loss of smell	43	1.7	0	0.0	2	0.9	17	3.1	19	3.7	5	1.4	0	0.0
Dyspnea	24	1.0	3	0.6	2	0.9	5	0.9	7	1.4	4	1.1	3	1.2
Myalgia (muscle aches)	206	8.3	10	1.9	12	5.1	59	10.9	68	13.1	46	12.8	11	4.6
Diarrhea	63	2.5	13	2.5	2	0.9	17	3.1	19	3.7	8	2.2	4	1.7
Nausea/vomiting	37	1.5	12	2.3	3	1.3	6	1.1	11	2.1	4	1.1	1	0.4
Others	191	7.7	40	7.8	16	6.8	32	5.9	51	9.9	29	8.1	23	9.5
Clinical features at the time of admission														
Time from symptom onset to PCR diagnosis, days, median (IQR)	2.0	(1.1)	2.2	(1.2)	2.0	(1.1)	2.0	(1.0)	1.9	(1.0)	2.0	(1.0)	1.8	(0.9)
Time from symptom onset to admission, days, median (IQR)	2.8	(1.6)	2.3	(1.5)	2.7	(1.6)	2.9	(1.5)	2.9	(1.6)	3.0	(1.6)	3.0	(1.6)
Cycle Threshold, median (IQR)														
Nucleocapsid (N)	19.0	(5.7)	18.8	(4.7)	19.3	(6.3)	20.5	(6.1)	20.4	(6.0)	20.1	(5.3)	20.6	(6.0)

Table S3. Characteristics of participants at enrollment (Jan-Mar 2022 (Omicron)) by age

**Characteristics**	**Jan-Mar 2022 (Omicron)**													
	**All patients**		**0-11 years**		**12-18 years**		**19-30 years**		**31-45 years**		**46-60 years**		**>60 years**	
	**n**	**%**	**n**	**%**	**n**	**%**	**n**	**%**	**n**	**%**	**n**	**%**	**n**	**%**
	**n=2447**		**n=516**		**n=234**		**n=544**		**n=518**		**n=360**		**n=242**	
<20	1162	53.9	280	64.2	112	54.3	233	46.5	258	47.7	172	50.9	107	45.8
20-30	974	45.2	137	31.4	75	36.4	248	49.5	262	48.5	146	43.2	106	45.4
>30	216	10.0	21	4.8	14	6.8	59	11.8	59	10.9	40	11.8	23	9.8
Envelope (E)	18.0	(5.4)	17.2	(4.3)	17.5	(5.9)	18.4	(6.3)	18.5	(5.7)	18.1	(5.6)	18.4	(6.0)
<20	1578	67.0	340	77.6	137	68.2	340	63.0	371	64.1	235	65.6	155	65.1
20-30	617	26.2	83	19.0	54	26.9	155	28.7	166	28.7	95	26.5	64	26.9
>30	159	6.8	15	3.4	10	5.0	45	8.3	42	7.3	28	7.8	19	8.0
RNA-dependent RNA polymerase (RdRp)	19.3	(5.4)	18.6	(4.4)	19.0	(5.6)	19.7	(5.9)	19.7	(5.8)	19.3	(5.4)	19.5	(5.5)

Physical symptoms information and laboratory analysis results of COVID-19 patients in the Omicron wave by age range. Data are presented as mean (SD), median (IQR), and range at $p < 0.05$ indicates statistical significance. SD, standard deviation; IQR, interquartile range; BT, body temperature; °C, degree Celsius, SARS-CoV-2 spike protein antibody titers, log_{10} transformed. Neutralizing antibody titers, log_{10} transformed.

Table S4. Independent factors of NCT of SARS-CoV-2 RNA by the multivariate Cox's proportional hazard model by age >18 years (Jul-Oct 2021 (Delta))

Factors	Survival analysis			
	Adjusted Hazard Ratio Exp(B)	95% CI for Exp(B)		*p-value*
		lower limit	detection limits	
Fever	0.75	0.70	0.81	<0.001*
Cough	0.84	0.81	0.87	<0.001*
Sore throat	1.20	1.13	1.27	0.048*
Rhinorrhea	1.20	1.14	1.26	0.027*
Productive sputum	0.99	0.95	1.04	0.955
Loss of taste	0.95	0.89	1.01	0.921
Loss of smell	0.81	0.77	0.85	<0001*
Dyspnea	0.87	0.83	0.91	0.034*
Myalgia	1.18	1.11	1.25	<0.001*
Diarrhea	1.37	1.24	1.52	0.503
Nausea/vomiting	1.09	0.96	1.24	0.154

Data are represented as Hazard Ratio (95% CI), the significant was determined by the Kaplan-Meier curves at $p < 0.05$.
NCT, negative conversion; SARS-CoV-2, severe acute respiratory syndrome coronavirus 2; CI, confidence interval

The Kaplan-Meier curves from multivariate Cox proportion estimating hazard rates between patients with and without symptom, adjusted age, sex and comorbidity were reported that patients who had systemic symptoms including fever (HR=0.75, 95% CI=0.70-0.80, p<0.001), cough (HR=0.84, 95% CI=0.81-0.87, p<0.001), and loss of smell (HR=0.81, 95% CI=0.77-0.85, p<0.001), had recovery rate greater than patients without these symptoms. On the other hand, patients with rhinorrhea (HR=1.20, 95% CI=1.14-1.26, p=0.027) or sore throat (HR=1.20, 95% CI=1.13-1.27, p=0.048) had recovery rate lower than another. The Kaplan-Meier curves revealed that fever, cough, and loss of smell had significantly prolonged NCT of SARS-CoV-2 RNA compared with the asymptomatic group (p<0.05).

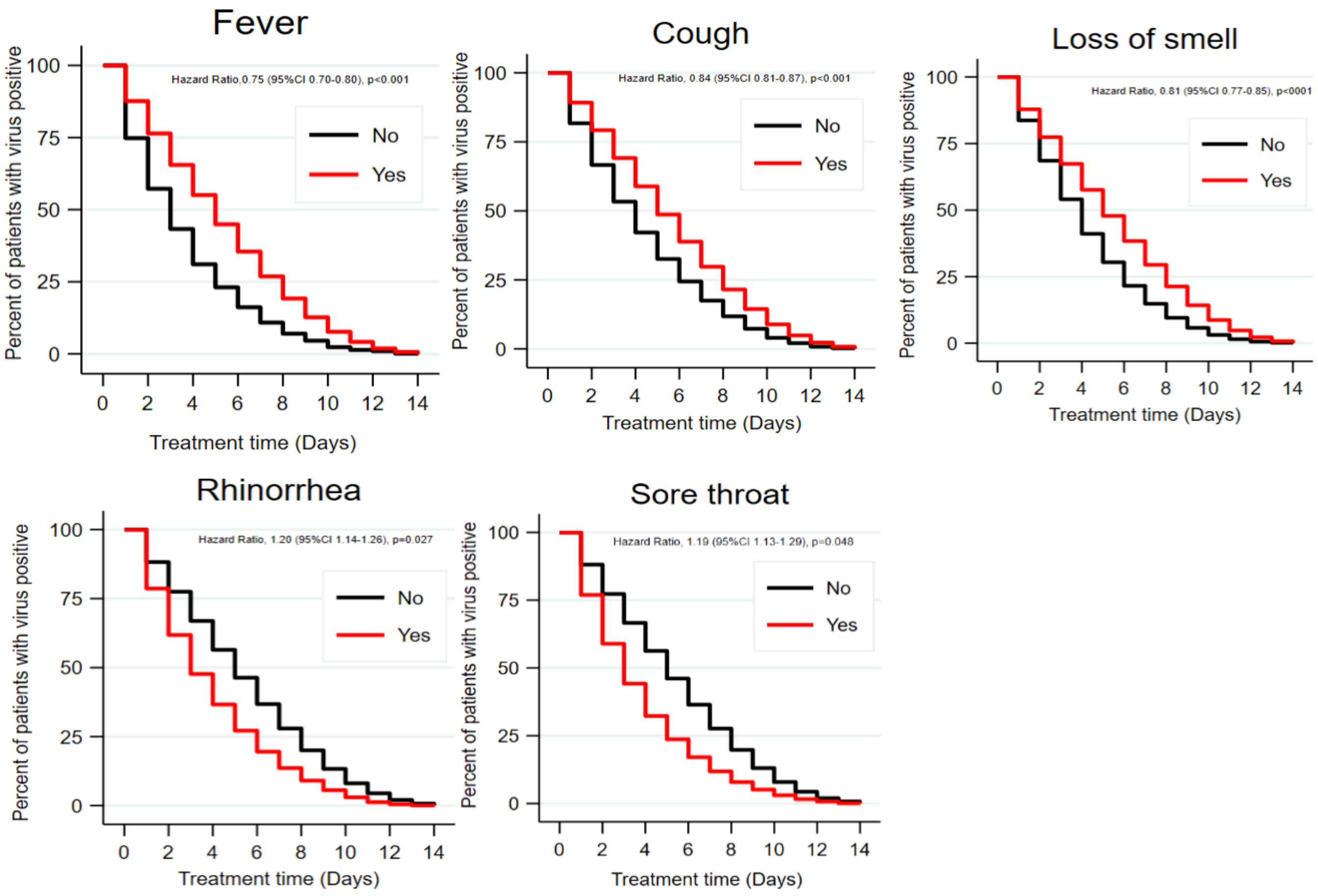

Figure S2. Kaplan-Meier estimate of time to symptom alleviation as reported by COVID-19-adapted FLU-PRO questionnaire (Jul-Oct 2021 (Delta))

Kaplan-Meier Estimate for treatment time (days) and percentage of patients infected with Delta in July-October 2022 by using self-reported symptoms questionnaire: patients had the symptom in red line and patients did not have symptom in black line.

Table S5. Characteristics of the first positive symptoms between the delta and omicron pandemics for people aged >18 years

vaccination	Jul - Oct 2021 (Delta) n=2029						Jan - Mar 2022 (omicron) n=1727					
	Total	Any symptoms		Cough, fever, anosmia, ageusia		*p-value*	Total	Any symptoms		Cough, fever, anosmia, ageusia		*p-value*
		n	%	n	%			n	%	n	%	
Unvaccinated	1069	957	89.5	931	87.1	0.033	109	96	88.1	48	44.0	0.005
0-14 days of 1 dose	635	559	88.0	543	85.5		33	26	78.8	15	45.5	
>14 days of 1 dose or 0-14 days of 2 doses	269	223	82.9	218	81.0		587	465	79.2	310	52.8	
>14 days of 2 dose or 0-14d days of 3 doses	56	37	66.1	36	64.3		511	402	78.7	265	51.9	
>14 days of 3 dose	-	-	-	-	-		475	384	80.8	302	63.6	
Reinfection pre-vaccinated	-	-	-	-	-		12	10	83.3	6	50.0	
p-value		0.011		0.034				0.145		0.341		

The significant was determined by Fisher extract's test at $p < 0.05$.

Table S6. Independent associations with symptom reporting between delta and omicron pandemic in aged>18 years

**Model**	**Jul-Oct 2021 (Delta) n=2029**				**Jan-Mar 2022 (Omicron) n=1727**			
	**Any symptoms**		**Cough, fever, anosmia, ageusia**		**Any symptoms**		**Cough, fever, anosmia, ageusia**	
	**OR (95% CI)**	***p-value***	**OR (95% CI)**	***p-value***	**OR (95% CI)**	***p-value***	**OR (95% CI)**	***p-value***
N gene (Ct)	0.77 (0.56-0.86)	0.000	0.90 (0.88-0.92)	0.000	1.00 (0.98-1.02)	0.915	0.95 (0.93-0.96)	0.000
Vaccination								
Unvaccinated	ref.		ref.					
0-14 days of 1 dose	0.79 (0.57-1.09)	0.155	0.80 (0.60-1.08)	0.153	0.34 (0.19-0.60)	0.000	1.01 (0.46-2.24)	0.979
>14 days of 1 dose or 0-14 days of 2 doses	0.51 (0.34-0.77)	0.001	0.60 (0.41-0.89)	0.012	0.65 (0.48-0.89)	0.006	1.28 (0.83-1.96)	0.265
>14 days of 2 dose or 0-14 days of 3 doses	0.25 (0.12-0.52)	0.000	0.28 (0.13-0.58)	0.001	0.58 (0.42-0.81)	0.001	1.26 (0.82-1.95)	0.287
>14 days of 3 doses	-	-	-	-	0.65 (0.46-0.92)	0.014	2.14 (1.38-3.32)	0.001
Reinfection pre-vaccinated	-	-	-	-	1.54 (0.35-6.78)	0.566	1.70 (0.50-5.76)	0.395

Data are represented as OR (95% CI), the significant was determined by binary logistic regression for age, sex, and comorbidity at $p < 0.05$. OR, Odds ratio; CI, confidence interval; N gene, nucleocapsid gene; Ct, cycle threshold

Table S7. Characteristics of the participants and geometric mean humoral immunogenicity assay responses according to anti-SARS-CoV-2 nucleocapsid protein and vaccine group

Characteristics	Anti-SARS-CoV-2 NP							Vaccine Group									
	**Total (n=337)**		**Negative (n=116)**		**Positive (n=221)**		*p-value*	**Total (n=495)**		**Unvaccinated (n=213)**		**ChAdOx1 (n=182)**		**CoronaVac (n=100)**		*p-value*
	n	%	n	%	n	%		n	%	n	%	n	%	n	%	
Sex																	
Male	123	36.5	43	37.1	80	36.2	0.483	179	36.2	81	38.0	64	35.2	34	34.0	0.723
Female	214	63.5	73	62.9	141	63.8		316	63.8	132	62.0	118	64.8	66	66.0	
Age, years, median (range)	43	(31-55)	39	(28-50.5)	44	(33-56)	0.012	43	(30-56)	40	(24-53)	49	(36-61)	39	(30-48)	<0.001
12-18	24	7.1	14	12.1	10	4.5	0.029	37	7.5	35	16.4	0	0.0	1	1.0	<0.001
19-45	167	49.6	58	50.0	109	49.3		238	48.1	94	44.1	79	43.7	65	65.0	
>45	146	43.3	44	37.9	102	46.2		220	44.4	84	39.4	102	56.4	34	34.0	
Presence of comorbidities																	
Hypertension	49	14.5	17	14.7	32	14.5	0.559	78	15.8	30	14.1	37	20.3	11	11.0	0.064
Dyslipidemia	26	7.7	11	9.5	15	6.8	0.265	38	7.7	7	3.3	23	12.6	8	8.0	0.001
Diabetes mellitus	25	7.4	7	6.0	18	8.1	0.304	38	7.7	15	7.0	19	10.4	4	4.0	0.136
Obesity	5	1.5	1	0.9	4	1.8	0.430	4	0.8	1	0.5	2	1.1	1	1.0	0.682
Asthma/COPD	18	5.3	4	3.5	14	6.3	0.441	28	5.7	10	4.7	13	7.1	5	5.0	0.769
Chronic heart disease	8	2.4	1	0.9	7	3.2	0.208	9	1.8	4	1.9	4	2.2	1	1.0	0.830
Others	67	19.9	24	20.7	43	19.5	0.446	93	18.8	49	23.0	33	18.1	11	11.0	0.037
Vaccine manufacturer																	
Unvaccinated	139	41.2	51	44.0	88	39.8	0.005	-	-	-	-	-	-	-	-	na
ChAdOx1	126	37.4	52	44.8	74	33.5		-	-	-	-	-	-	-	-	
CoronaVac/ BBIBP-CorV	72	21.4	13	11.2	59	26.7		-	-	-	-	-	-	-	-	
Vaccine doses administered																	
Unvaccinated	139	41.2	51	44.0	88	39.8	<0.001	213	43.0	213	100.0	0	0.0	0	0.0	<0.001
1 dose	102	30.3	48	41.4	54	24.4		158	31.9	0	0.0	143	78.6	15	15.0	

Table S7. Characteristics of the participants and geometric mean humoral immunogenicity assay responses according to anti-SARS-CoV-2 nucleocapsid protein and vaccine group

Characteristics	Anti-SARS-CoV-2 NP							Vaccine Group									
	**Total (n=337)**		**Negative (n=116)**		**Positive (n=221)**		*p-value*	**Total (n=495)**		**Unvaccinated (n=213)**		**ChAdOx1 (n=182)**		**CoronaVac (n=100)**		*p-value*
	n	%	n	%	n	%		n	%	n	%	n	%	n	%	
2 doses	76	22.6	15	12.9	61	27.6		100	20.2	0	0.0	15	8.2	85	85.0	
3 doses	20	5.9	2	1.7	18	8.1		24	4.8	0	0.0	24	13.2	0	0.0	
SARS-CoV-2																
seronegative	-	-	-	-	-	-	na	116	34.4	51	36.7	52	41.3	13	18.1	0.002
seropositive	-	-	-	-	-	-		221	65.6	88	63.3	74	58.7	59	81.9	
Immunoglobulin G (mg/ml); GMT, (95%CI)	809.8	(696-942.3)	1079	(918.6-1268)	470.9	(351.2-631.5)	<0.001	797.3	(702.9-904.4)	587	(463.9-742.7)	1094	(950.7-1260)	884.9	(701-1117)	0.002
Neutralizing antibody; GMT (95%CI)	97.5	(94.7-98.2)	70.7	(61.3-81.6)	91.6	(89.1-94.2)	<0.001	97.4	(94.6-98.0)	78.9	(74.4-83.6)	94.8	(92.2-97.5)	93.3	(90.2-96.4)	<0.001
Days since most recent positive viral test, median (range)	89	(71.5-120)	102	(79-124)	84	(69-114)	0.007	98	(81-107)	99	(94-120)	91	(70-100)	98	(84-107)	<0.001
Days since receipt of first vaccine dose, median (range)	135	(114-159)	135	(119-162)	135	(113-159)	0.884	133	(117-151)	-	-	133	(117-150)	128	(114-198)	0.076
Days since receipt of second vaccine dose, median (range)	132	(63-177)	59	(34.5-171)	142	(92-181)	0.004	136	(65.5-179)	-	-	55	(33-117)	170	(136-187)	<0.001
Days since receipt of third vaccine dose, median (range)	109	(88-125)	103	(102-104)	111	(88-125)	0.739	118	(109-137)	-	-	118	(109 - 137)	-	-	na
Cycle Threshold (viral load at the time of entering HI)																
Nucleocapsid (N), median (range)	20.0	(17.2-23.8)	20.9	(17.9-26.5)	19.2	(16.8-22.9)	0.049	20.2	(17.5-24.6)	20.4	(17.5-25.8)	20.1	(17.4-24.2)	20.0	(16.8-24.6)	

Table S7. Characteristics of the participants and geometric mean humoral immunogenicity assay responses according to anti-SARS-CoV-2 nucleocapsid protein and vaccine group

Characteristics	Anti-SARS-CoV-2 NP							Vaccine Group									
	Total (n=337)		Negative (n=116)		Positive (n=221)		*p-value*	Total (n=495)		Unvaccinated (n=213)		ChAdOx1 (n=182)		CoronaVac (n=100)		*p-value*
	n	%	n	%	n	%		n	%	n	%	n	%	n	%	
<20	153	50.5	45	42.1	108	55.1	0.004	212	48.0	93	45.8	81	49.7	38	50.0	0.587
20-30	125	41.3	46	43.0	79	40.3		192	43.4	95	46.8	68	41.7	29	38.2	
>30	25	8.3	16	15.0	9	4.6		38	8.6	15	7.4	14	8.6	9	11.8	
Envelope (E), median (range)	16.8	(13.4-21.1)	17.7	(14.0-23.0)	16.3	(13.2-19.9)	0.040	17.0	(13.6-21.6)	17.2	(13.5-22.2)	16.5	(13.5-21.1)	17.1	(14.2-22.3)	
<20	231	71.3	71	63.4	160	75.5	0.028	325	69.3	142	69.3	116	69.5	67	69.1	0.286
20 - 30	80	24.7	33	29.5	47	22.2		128	27.3	58	28.3	47	28.1	23	23.7	
>30	13	4.0	8	7.1	5	2.4		16	3.4	5	2.4	4	2.4	7	7.2	
RNA-dependent RNA polymerase (RdRp), median (range)	21.5	(18.4-25.1)	21.8	(19.1 - 28.2)	20.9	(18.0-24.2)	0.004	21.7	(18.7-26.1)	21.9	(18.7-27.2)	21.2	(18.6-25.4)	21.6	(18.8-26.1)	0.778

Categorical and continuous data of characteristics of the participants and geometric mean humoral immunogenicity assay responses presented as n (%) and median (range)at $p < 0.05$ indicates statistical significance. COPD, chronic obstructive pulmonary disease; GMC, geometric mean concentration; ChAdOx1, AstraZeneca vaccine; CoronaVac, Sinovac vaccine; BBIBP-CorV, Sinopharm vaccine; SARS-CoV-2, the severe acute respiratory syndrome-coronavirus-2; NP, nucleocapsid protein; CI, confident intervals; Ct, cycle threshold; na, not available.

Table S8. Anti-RBD IgG and sVNT geometric mean concentration (GMC) among unvaccinated, ChAdOx1, and CoronaVac

**Vaccinations/Day**	**Unvaccinated**			**ChAdOx1**									**CoronaVac**						*p-value*
	**25-56 days**	**56-84 days**	**>84 days**	**25-56 days**			**56-84 days**			**>84 days**			**25-56 days**		**56-84 days**		**>84 days**		
				**1 dose**	**2 doses**	**3 doses**	**1 dose**	**2 doses**	**3 doses**	**1 dose**	**2 doses**	**3 doses**	**1 dose**	**2 doses**	**1 dose**	**2 doses**	**1 dose**	**2 doses**	
Number of objectives	22	42	149	19	10	10	40	12	11	49	18	13	9	10	12	14	16	39	
SARS-CoV-2 IgG																			0.022
GMT of IgG	299.5	597.2	645.1	1655	1440	1538	1025	2075	1674	822.9	945.3	886.1	566.8	897.6	789.1	712.1	1174	974.5	
Lower 95% CI	140.9	339.6	488.4	1217	889	861.7	742.6	1057	1147	626.4	567.4	588.2	252	450.6	305.5	315.3	638.4	684.9	
Upper 95% CI	636.6	1050	852	2251	2332	2747	1414	4074	2443	1081	1575	1335	1275	1788	2038	1608	2158	1386	
p-value		0.044			*ns*			*ns*			*ns*		*ns*		*ns*		*ns*		
SARS-CoV-2 sVNT																			<0.0001
GMT of sVNT	74.3	75.7	80.5	97.8	97.4	97.3	90.2	97.4	98.0	95.2	95.1	96.4	92.7	92.8	87.1	86.8	95.6	97.2	
Lower 95% CI	62.5	63.3	75.6	97.4	96.6	96.4	79.8	96.6	97.7	93.9	93.0	94.8	87.3	86.5	73.7	71.1	92.6	96.6	
Upper 95% CI	88.3	90.6	85.6	98.2	98.3	98.2	102.0	98.2	98.3	96.6	97.2	97.9	98.5	99.4	103.0	105.9	98.8	97.7	
p-value		*ns*			*ns*			*ns*			*ns*		*ns*		*ns*		*ns*		

Relation of vaccinations ChAdOx1 (AstraZeneca), CoronaVac (Sinovac), Unvaccinated and laboratory findings of all confirmed COVID-19 patients. Presented as vaccinated 25-56 days, 56-84 days, >84 days and range at $p < 0.05$ indicates statistical significance. SARS-CoV-2 IgG quantitative immunological test. By examining the ability to prevent the severity of symptoms when infected. SARS-CoV-2 sVNT Immunity test by examining the ability to inhibit invasion and viral proliferation using the Surrogate virus Neutralization test (sVNT). SARS_CoV-2, the severe acute respiratory syndrome-Coronavirus-2; IgG, immunoglobulin G; GMT, geometric mean titer; sVNT, the surrogate virus neutralization test

Table S9. SARS-CoV-2 sVNT geometric mean concentration (GMC) among unvaccinated, ChAdOx1, and CoronaVac by Wuhan and Delta variants

Days/Age	25-56 days				56-84 days				>84 days				*p-value*
	Age <45		Age ≥45		Age <45		Age ≥45		Age <45		Age ≥45		
	Wuhan	Delta	Wuhan	Delta	Wuhan	Delta	Wuhan	Delta	Wuhan	Delta	Wuhan	Delta	
**Unvaccinated**													<0.0001
Number of subjects	12	9	10	8	27	15	26	15	88	60	84	59	
GMT of sVNT	72.1	77.4	18.9	32.2	69.5	88.3	29.0	47.6	76.5	86.6	37.0	48.0	
Lower 95% CI of GMT	55.2	59.8	3.2	9.6	53.1	76.5	15.3	27.3	69.6	81.2	26.8	34.8	
Upper 95% CI of GMT	94.1	100.2	113.0	108.3	91.0	101.9	55.0	83.1	84.1	92.4	51.1	66.1	
p-value	0.021		0.034		*ns*		*ns*		0.015		0.004		
**ChAdOx1**													<0.0001
Number of subjects	15	24	15	24	30	33	29	33	35	45	35	45	
GMT of sVNT	97.2	97.8	75.4	89.1	88.0	97.5	63.8	79.7	94.6	96.0	47.9	71.1	
Lower 95% CI of GMT	96.4	97.5	61.7	84.0	74.6	97.1	47.3	69.8	93.0	94.9	35.7	59.3	
Upper 95% CI of GMT	97.9	98.2	92.2	94.6	103.8	97.9	86.1	91.0	96.2	97.2	64.3	85.2	
p-value	0.032		ns		0.028		0.007		0.0001		0.001		
**CoronaVac**													<0.0001
Number of subjects	12	11	12	11	18	8	18	8	35	15	35	15	
GMT of sVNT	84.6	94.2	24.3	63.7	94.6	94.4	63.5	56.3	94.0	96.3	71.2	68.6	
Lower 95% CI of GMT	66.8	90.5	8.6	42.3	91.1	88.0	43.1	24.4	89.0	94.6	54.0	51.7	
Upper 95% CI of GMT	107.1	98.1	68.6	95.9	98.2	101.2	93.4	129.9	99.3	97.9	93.7	91.2	
p-value	0.001		0.013		0.038		0.009		0.007		0.027		

Represents the mean sVNT geometric mean concentration (GMC) of unvaccinated, AstraZeneca COVID-19 (ChAdOx1) vaccinated, and Sinovac COVID-19 (CoronaVac) vaccinated patients. Comparing each vaccination group and each age group. During the Wuhan and Delta outbreaks, there was no difference in sVNT values. In addition to the AstraZeneca COVID-19 (ChAdOx1) vaccinated before infection >84 days in the age range ≥45 years, were found statistical differences. SARS-CoV-2, the severe acute respiratory syndrome-coronavirus-2; sVNT, the surrogate virus neutralization test; GMC, geometric mean concentration; CI, confidence interval; ns, not statistically significant.

Table S10. Anti-RBD IgG and sVNT geometric mean concentration (GMC) among unvaccinated, ChAdOx1, and CoronaVac by age

Vaccinations/Age	Unvaccinated		ChAdOx1		CoronaVac		*p-value*
	**Age<45**	**Age≥45**	**Age<45**	**Age≥45**	**Age<45**	**Age≥45**	
Number of objectives	129	84	80	102	66	34	
SARS-CoV-2 IgG							<0.001
GMT of IgG	486	784.2	830.8	1418	822.4	1020	
Lower 95% CI of GMT	350.1	570.5	682.4	1188	608.8	700.8	
Upper 95% CI of GMT	674.6	1078	1012	1692	1111	1484	
p-value	0.001		0.004		*ns*		
SARS-CoV-2 sVNT							<0.001
GMT of sVNT	74.5	85.9	92.5	96.9	92.3	95.2	
Lower 95% CI of GMT	68.3	81.2	87.1	96.4	87.8	93.3	
Upper 95% CI of GMT	81.4	90.9	98.3	97.5	97.0	97.0	
p-value	0.034		*ns*		*ns*		

Relation of vaccination ChAdOx1 (AstraZeneca), CoronaVac (Sinovac), Unvaccinated and laboratory findings of all confirmed COVID-19 patients. Presented patients age <45 and ≥45 years range at $p<0.05$ indicates statistical significance. SARS-CoV-2 IgG quantitative immunological test. By examining the ability to prevent the severity of symptoms when infected. SARS-CoV-2 sVNT Immunity test by examining the ability to inhibit invasion and viral proliferation using the Surrogate virus Neutralization test (sVNT). Anti-RBD IgG, anti-receptor binding domain immunoglobulin G; IgG, immunuglobulin G; SARS-CoV-2, the severe acute respiratory syndrome-coronavirus-2; sVNT, the surrogate virus neutralization test; GMT, Geometric mean titer; ns, not statistically significant.

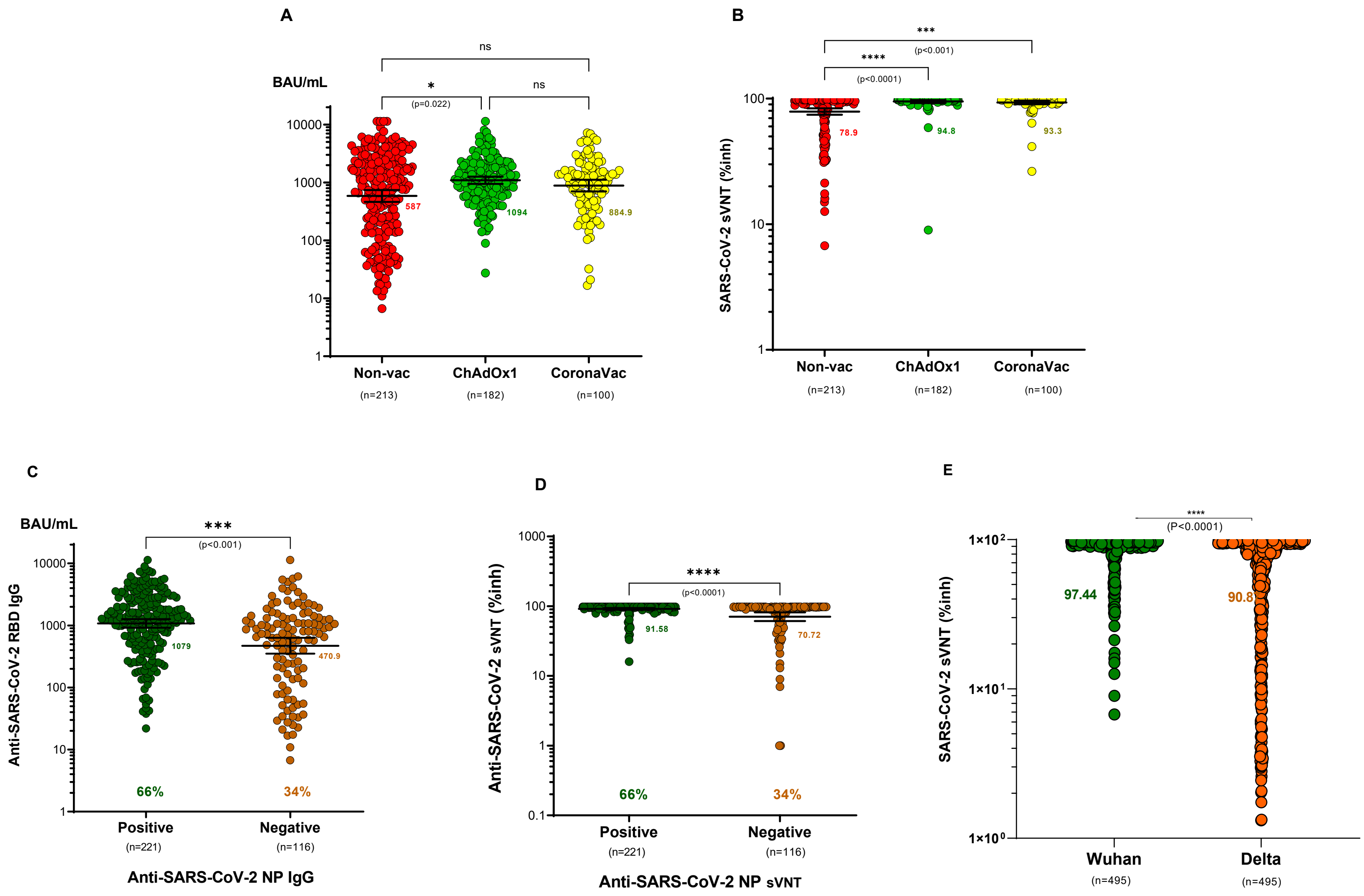

Figure S3. Plasma anti-immunoglobulin G (IgG) and antibody (sVNT) levels against SARS

A Anti-SARS-CoV-2 spike receptor binding domain (RBD) IgG antibodies in plasma of unvaccinated cases (n=213), ChAdOx1 nCoV-19 vaccinated cases (n=182), and CoronaVac vaccinated cases (n=100) were measured by chemiluminescent microparticle immunoassay.

B Anti-SARS-CoV-2 neutralizing antibodies in plasma of unvaccinated cases (n=213), ChAdOx1 nCoV-19 vaccinated cases (n=182), and CoronaVac vaccinated cases (n=100) were measured by surrogate virus neutralization test (sVNT).

C Anti-SARS-CoV-2 spike RBD IgG antibodies in plasma of positive anti-SARS-CoV-2 nucleocapsid protein cases (n=221) and negative anti-SARS-CoV-2 nucleocapsid protein cases (n=116) were determined by chemiluminescent microparticle immunoassay and SARS-CoV-2 IgG II Quant for use with ARCHITECT; Abbott Laboratories.

D Anti-SARS-CoV-2 neutralizing antibodies in plasma of positive anti-SARS-CoV-2 nucleocapsid protein cases (n=221) and negative anti-SARS-CoV-2 nucleocapsid protein cases (n=116) were measured by surrogate virus neutralization test (sVNT) and SARS-CoV-2 IgG II Quant for use with ARCHITECT; Abbott Laboratories.

E Anti-SARS-CoV-2 neutralizing antibodies in plasma of wild-type (Wuhan) strain infection cases (n=495) and Delta (B.1.617.2) strain infection cases (n=495) were measured by surrogate virus neutralization test (sVNT).

Data information

A-B: Statistical comparisons were determined by two-tailed Kruskal-wallis test.

C-E: Statistical comparisons were determined by two-tailed Mann-Whitney U-test.

In all graphs, error bars denote median and interquartile range.

References

1. World Health Organization Thailand. COVID-19 - WHO Thailand situation reports. Available online: https://www.who.int/thailand/emergencies/novel-coronavirus-2019/situation-reports (accessed on 5 July 2022).
2. Department of Disease Control. Corona virus disease (COVID-19). Available online: https://ddc.moph.go.th/covid19-dashboard/ (accessed on 8 June 2022).
3. Kalin, A.; Javid, B.; Knight, M.; Inada-Kim, M.; Greenhalgh, T. Direct and indirect evidence of efficacy and safety of rapid exercise tests for exertional desaturation in Covid-19: a rapid systematic review. *Syst. Rev.* **2021**, *10*, 77, doi:10.1186/s13643-021-01620-w.
4. Sirijatuphat, R.; Suputtamongkol, Y.; Angkasekwinai, N.; Horthongkham, N.; Chayakulkeeree, M.; Rattanaumpawan, P.; Koomanachai, P.; Assanasen, S.; Rongrungruang, Y.; Chierakul, N.; et al. Epidemiology, clinical characteristics, and treatment outcomes of patients with COVID-19 at Thailand's university-based referral hospital. *BMC Infect. Dis.* **2021**, *21*, 382-382, doi:10.1186/s12879-021-06081-z.
5. Aranha, C.; Patel, V.; Bhor, V.; Gogoi, D. Cycle threshold values in RT-PCR to determine dynamics of SARS-CoV-2 viral load: An approach to reduce the isolation period for COVID-19 patients. *J. Med. Virol.* **2021**, *93*, 6794-6797, doi:10.1002/jmv.27206.
